# LSD1 modulates the non-canonical integrin β3 signaling pathway in non-small cell lung carcinoma cells

**DOI:** 10.1038/s41598-017-09554-x

**Published:** 2017-08-31

**Authors:** So-Young Lim, Iris Macheleidt, Priya Dalvi, Stephan C. Schäfer, Martin Kerick, Luka Ozretić, Sandra Ortiz-Cuaran, Julie George, Sabine Merkelbach-Bruse, Jürgen Wolf, Bernd Timmermann, Roman K. Thomas, Michal R. Schweiger, Reinhard Buettner, Margarete Odenthal

**Affiliations:** 1 Institute of Pathology, University Hospital of Cologne, 50931 Cologne, Germany; 2 The Center for Molecular Medicine Cologne (CMMC), 50931 Cologne, Germany; 3 Center of Integrative Oncology, University Clinic of Cologne and Bonn, 50937 Cologne, Germany; 4 Functional Epigenomics, University of Cologne, 50931 Cologne, Germany; 5 Department of Translational Genomics, Medical Faculty, University of Cologne, 50931 Cologne, Germany; 6 Centre Léon Bérard, 69008 Lyon, France; 7 Lung Cancer Group Cologne, University Hospital of Cologne, 50937 Cologne, Germany; 8 Clinic for Internal Medicine, University Hospital of Cologne, 50937 Cologne, Germany; 9 Max Planck Institute for Molecular Genetics, 14195 Berlin, Germany; 10 German Cancer Research Center, German Cancer Consortium (DKTK), 69120 Heidelberg, Germany

## Abstract

The epigenetic writer lysine-specific demethylase 1 (LSD1) is aberrantly upregulated in many cancer types and its overexpression correlates with poor survival and tumor progression. In this study, we analysed LSD1 function in non-small cell lung cancer adenocarcinomas. Expression profiling of 182 cases of lung adenocarcinoma proved a significant correlation of LSD1 overexpression with lung adenocarcinoma progression and metastasis. KRAS-mutated lung cancer cell clones were stably silenced for LSD1 expression. RNA-seq and comprehensive pathway analysis revealed, that genes related to a recently described non-canonical integrin β3 pathway, were significantly downregulated by LSD1 silencing. Hence, invasion and self-renewal capabilities were strongly decreased. Notably, this novel defined LSD1/integrin β3 axis, was also detected in human lung adenocarcinoma specimens. Furthermore, the linkage of LSD1 to an altered expression pattern of lung-lineage specific transcription factors and genes, which are involved in alveolar epithelial differentiation, was demonstrated. Thus, our findings point to a LSD1-integrin β3 axis, conferring attributes of invasiveness and tumor progression to lung adenocarcinoma.

## Introduction

Lung cancer is the leading cause of cancer-related deaths worldwide. The high mortality associated with lung cancer is partly due to metastasis before surgical removal of the primary tumor^1^. Lung cancer is classified into non-small cell lung cancer (NSCLC), small cell lung cancer (SCLC) and pulmonary carcinoids. NSCLC comprises the majority of lung cancers and is further divided into adenocarcinoma (AC), squamous cell carcinoma (SQ) and large cell neuroendocrine carcinoma (LCNEC)^2^. Each subtype of lung cancer has been shown to derive from different cells of origin and carries distinct somatic genetic alterations. SCLC originates from neuroendocrine cells and harbors typically two genetic alterations that inactivate both alleles of TP53 and RB^3^, whereas AC develops from transformed alveolar epithelial cells and often harbor EGFR mutations, KRAS mutations, or EML4-ALK fusions^2,4^.

Recent reports have shown that in a wide variety of epithelial cancers including lung cancer the expression of the integrin α*v*β3 complex is associated with a poor outcome and higher incidences of metastasis^5,6^. Integrin complexes provide a mechanistic link between the actin cytoskeleton and the extracellular matrix, leading to morphogenic changes and cell migration. Interestingly, Seguin *et al*. showed that activation of the integrin α*v*β3 complex by non-canonical ligand galectin-3 binding recruits KRAS to the tumor cell plasma membrane, which in turn results in a prominent RalB and NF-κB activation^7^. This non-canonical integrin α*v*β3-KRAS-NF-κB signal axis serves as a driving force for breast, lung and pancreatic carcinomas with stem-like properties, that are highly aggressive and resistant to receptor tyrosine kinase inhibitors such as erlotinib^7^.

Epigenetic dysregulation has been demonstrated to be involved in the initiation and progression of many cancer types^8,9^. In NSCLC, histone modifications such as histone acetylation and methylation are crucial prognostic indicators for tumor progression and malignancy^9^. The lysine specific demethylase 1, LSD1, also known as KDM1A, is a key epigenetic writer and specifically demethylates mono- and di- methylated histone H3K4 and H3K9^10,11^. LSD1 is aberrantly upregulated in many human cancer types, such as prostate, breast and lung carcinoma as well as neuroblastoma and leukemia^12–16^. Importantly, cancer-related LSD1 overexpression is associated with increased cell proliferation, invasion and migration underscoring the therapeutic potential of LSD1 inhibition^13,16,17^. In a proliferation screen of cell lines representing various tumor types, acute myeloid leukemia (AML) and SCLC cell lines have been shown to be particularly sensitive to pharmacological LSD1 inhibitors, resulting in cytostatic growth inhibition and advanced differentiation^15,18^.

In the present study, we demonstrate that LSD1 is specifically upregulated in poorly differentiated and metastasized lung AC. Surprisingly, LSD1 knockdown induced a broad transcriptome change as demonstrated by comprehensive RNA-seq gene expression profiling. In particular, LSD1 knockdown resulted in downregulation of genes, which are involved in the previously described non-canonical integrin β3 affecting the stem-like phenotype with invasion and self-renewal capacities^7^. Mechanistically, LSD1 was critical for integrin β3 and galectin-3 expression. Furthermore, overexpression of LSD1 significantly correlated with integrin β3 expression in lung AC. Therefore, our findings provide a rationale to overcome cancer stemness and metastasis by targeting LSD1 in NSCLC in future therapeutic strategies.

## Results

### High LSD1 expression indicates lung cancer malignancy

**Figure 1:**
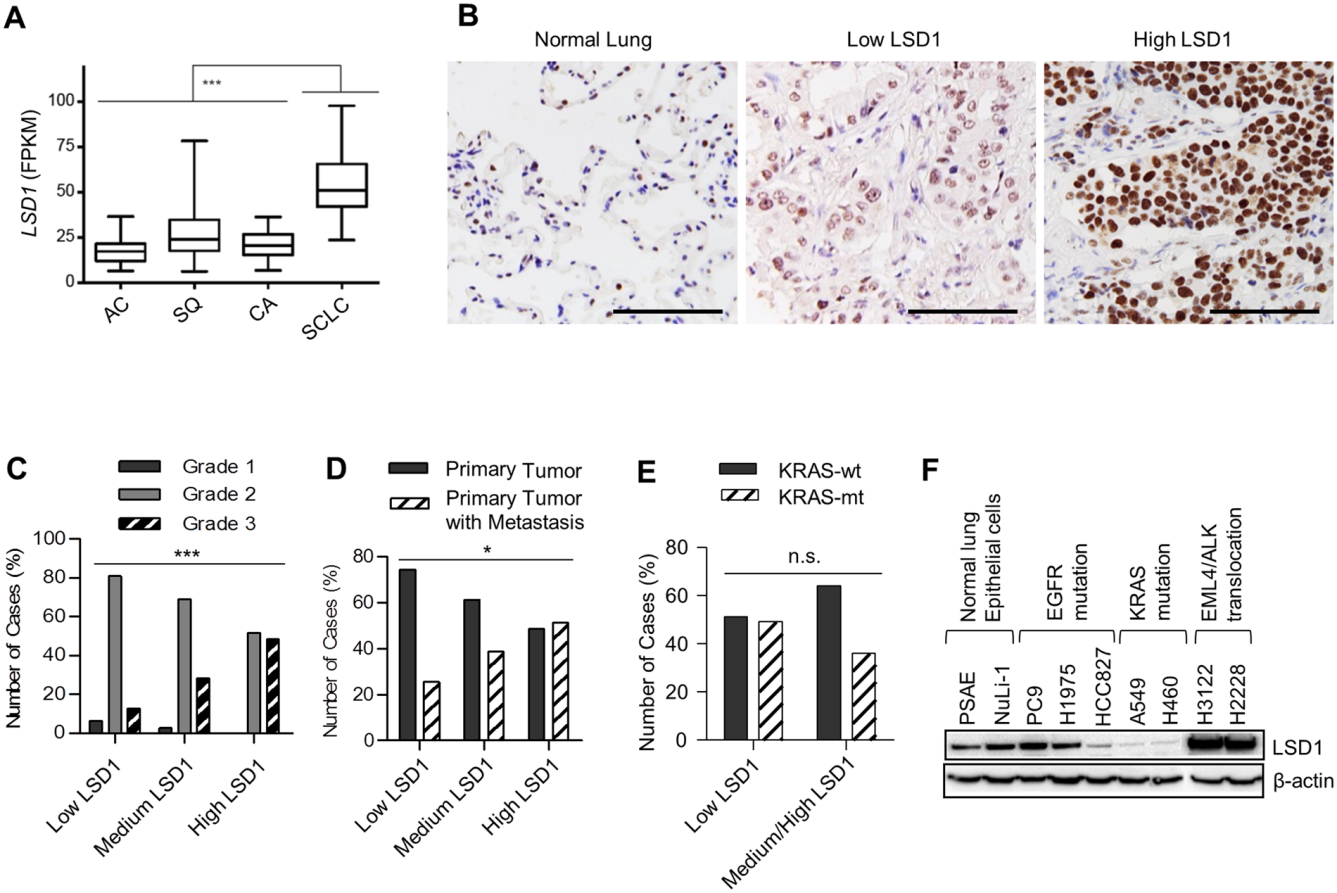
Overexpression of LSD1 in high grade and metastasized lung adenocarcinoma. (**A**) The bar graph with 5–95 percentiles showing *LSD1* mRNA expression in different types of lung tumors as determined by previously published transcriptome sequencing data for AC = lung adenocarcinoma (n = 40)^19,20^, SQ = squamous lung carcinoma (n = 9)^19^, CA = carcinoid (n = 69)^21^, SCLC = small cell lung cancer (n = 80)^3^. *LSD1* expression is represented by Fragments Per Kilobase of exon per Million fragments mapped (FPKM). Original data are provided in Supplementary Table S1. Mann-Whitney U test was used to calculate the statistical significance. ***P < 0.001. (**B**) Representative images of immunostainings with LSD1 antibodies on TMAs including 182 NSCLC AC and 11 non-tumor lung tissues. Immunostaining showed only moderate LSD1 expression in normal lung tissues, but elevated LSD1 expression in NSCLC-AC. The LSD1 expression in NSCLC-AC was classified using a low, medium and high immunostaining score as shown exemplarily for low and high LSD1 expression. Scale bars indicate 100 µm. (**C–E**) LSD1 expression analysed by immunohistochemistry on 182 lung adenocarcinomas was correlated with tumor grades (**C**), lymph node metastasis (**D**) and KRAS mutation (**E**). Chi-Square tests were used to calculate the statistical significance for linear-by-linear association. *P < 0.05, ***P < 0.001 and *n.s*. = not significant. Original data for C-E can be found in Supplementary Table S2. **(F)** LSD1 expression in non-small cell lung cancer cell lines with various mutational background determined by immunoblot.

We first determined LSD1 expression in the previously published transcriptome sequencing data comprising 198 lung cancer specimens (Fig. 1A and Supplementary Table S1)^3,19–21^. Interestingly, the highest LSD1 expression levels were detected in SCLC, which is associated with the worst prognosis^22^ (Fig. 1A). Albeit LSD1 expression was lower in AC than in other lung cancer types, immunohistology on lung AC compared with non-tumorous lung tissues implies that LSD1 is also overexpressed in AC (Fig. 1B). To determine the implication of LSD1 expression in NSCLC AC, LSD1 expression levels were assessed by comprehensive immunohistochemistry analyses of 182 lung AC specimens (Fig. 1B and Table 1). All ACs were derived from patients who did not receive any preoperative or postoperative treatment and were classified according to the tumor grade, lymph node metastasis, and the KRAS and EGFR mutational status (Table 1). A markedly more intense LSD1 staining was observed in poorly differentiated, high grade AC (Fig. 1B and C)^23^. Moreover, LSD1 expression was significantly higher in primary AC with lymph node metastasis than the ones which had not developed metastasis (Fig. 1D). In addition, in NSCLC specimens with high LSD1 expression, we observed a slightly less KRAS mutation frequency. However, no statistical significance was found for an inverse correlation between the KRAS mutation status and LSD1 expression (Fig. 1E). Further analyses of LSD1 protein expression on various NSCLC cell lines also suggested a possible inverse link between LSD1 expression and constitutive KRAS activation by mutation (Fig. 1F).

**Table 1:** Patient Characteristics.

Characteristic	No. of Patients (N = 182)	%
**Age**		
<70	95	52
≥70	87	48
**Sex**		
Male	110	61
Female	70	39
**Grade**		
1	7	4
2	114	69
3	45	27
**Nodal status**		
Negative	113	62
Positive	69	38
**KRAS-mutation status**		
KRAS wild type	104	59
KRAS mutation	73	41
**EGFR-mutation status**		
EGFR wild type	161	90
EGFR mutation	18	10

### LSD1 is critical for an invasive phenotype of A549 cells by regulating the non-canonical integrin pathway

**Figure 2:**
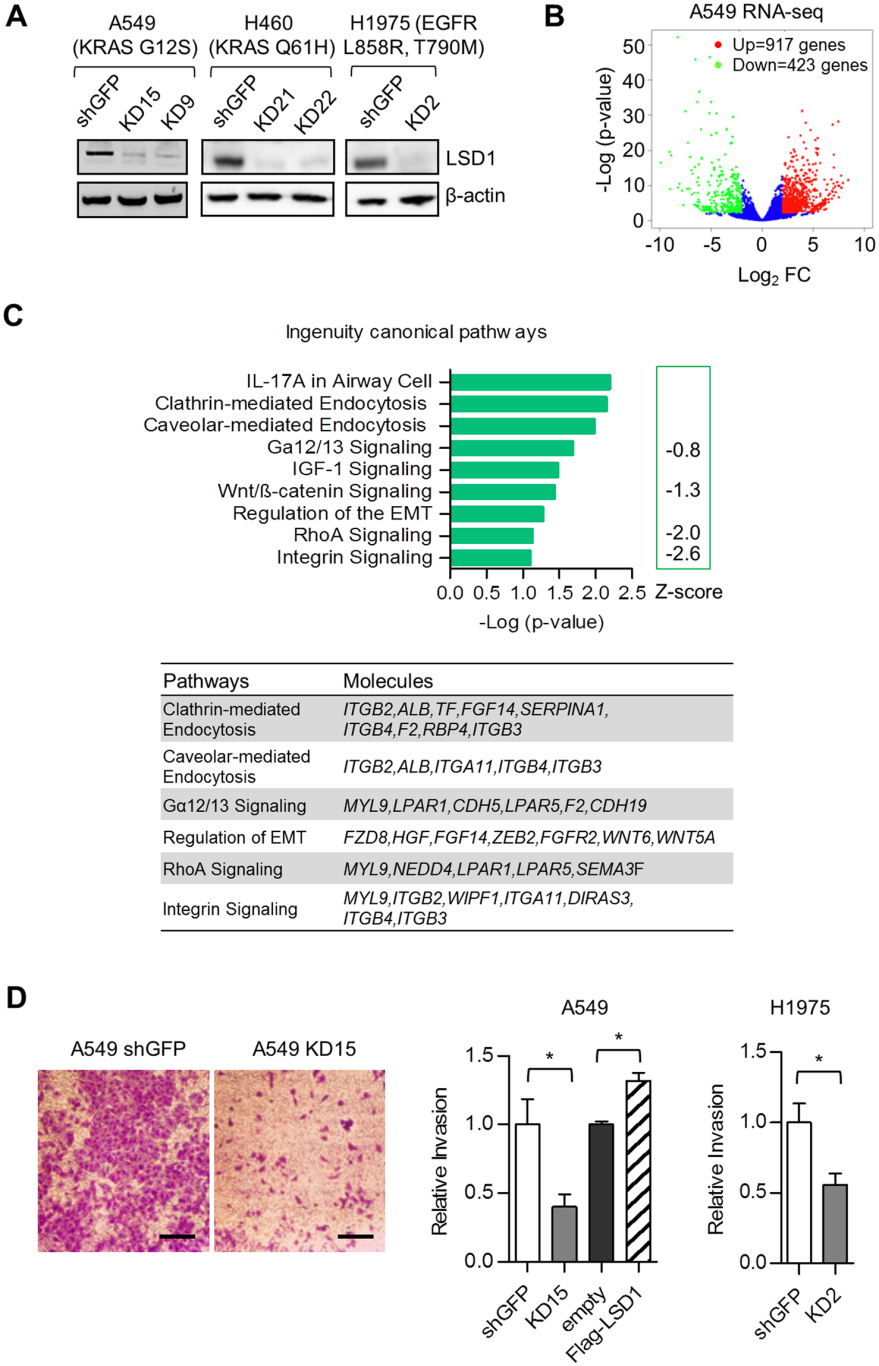
LSD1 is critical for the invasive phenotype of A549 cells by regulating the integrin pathway. (**A**) Knockdown efficiency of LSD1 in cells stably expressing LSD1 shRNA (KD9-clone 9, KD15-clone 15 in A549 cells, KD21-clone 21, KD22-clone 22 in H460 cells and KD2-clone 2 in H1975 cells) relative to shGFP control cells was shown by western blot. LSD1 protein levels were shown by western blot and the β-actin protein was used as loading controls. Data are representative of three independent experiments. (**B**) The RNA-seq of A549 cells. Differentially regulated genes after LSD1 shRNA knockdown (shGFP vs KD15) were shown by the Volcano blot. 917 up-regulated genes (p < 0.05 & log_2_- fold change > 2) are shown in red, while 423 down-regulated genes are shown in green (p < 0.05 & log_2_-fold change < −2). (**C**) The bar graph showing the representative canonical pathways affected by LSD1 knockdown. The Ingenuity activation z-score is a statistical measure of the match between expected relationship direction from literature and observed gene expression in RNA-seq. The z-score of Integrin Signaling (z-score <−2) predicts the significant inhibition state of the integrin signaling pathway in A549 shLSD1 (KD15) relative to A549 shGFP. The differentially regulated genes in the pathways are shown in a table (right panel). (**D**) Representative images of invasion assay of A549 cells expressing GFP shRNA control or LSD1 shRNA (left panel). Bar graphs showing the quantification of Crystal Violet staining of Boyden Chamber transwell filters (right panel). The bar graphs represents the mean ± SEM for n = 4 (A549) and n = 5 (H1975). *P < 0.05 (Student’s t-test). Scale bars, 50 µm.

To investigate the function of LSD1 in NSCLC, stable LSD1 knockdown (KD) cells from A549 lung AC cell line were generated using retroviral transduction of a shLSD1 construct primarily tested to be highly efficient. Two monoclonal cell populations (KD9 and KD15) (Fig. 2A) were established and used for RNA-seq experiment. Although in the A549 KD cells LSD1 was not among the top 100 downregulated genes, RNA-seq analysis revealed that LSD1 silencing in A549 cells resulted in a dramatic change in the transcriptome profile. A total of 917 genes were upregulated, whereas 423 genes were downregulated (Fig. 2B and Supplementary Table S3). Ingenuity pathway analysis (IPA) showed that pathways such as clathrin- or caveolar-mediated endocytosis, Gα12/13, RhoA and integrin signaling, which are all associated with invasive migration, were affected by LSD1 silencing (Fig. 2C). The influence of LSD1 on altered expression profiles of invasion and migration related pathways, were confirmed by transwell migration assays demonstrating impaired invasive capacities of LSD1 silenced A549 cells (Fig. 2D). In order to validate these findings, we established additional LSD1 KD clones from the KRAS Q61H mutated H460 and H1975 carrying the EGFR L858R, T790M mutation (Fig. 2A). Similarly to LSD1 silencing of A549 cells, LSD1 knockdown decreased invasion capacity of H1975 cells (Fig. 2D). Surprisingly, LSD1 silencing had only a moderate effect on cell growth and no signs of apoptosis were observed in A549 cells (Supplementary Fig. S1A).

### LSD1 contributes to self-renewal by modulating integrin β3-KRAS-NF-κB pathway

**Figure 3:**
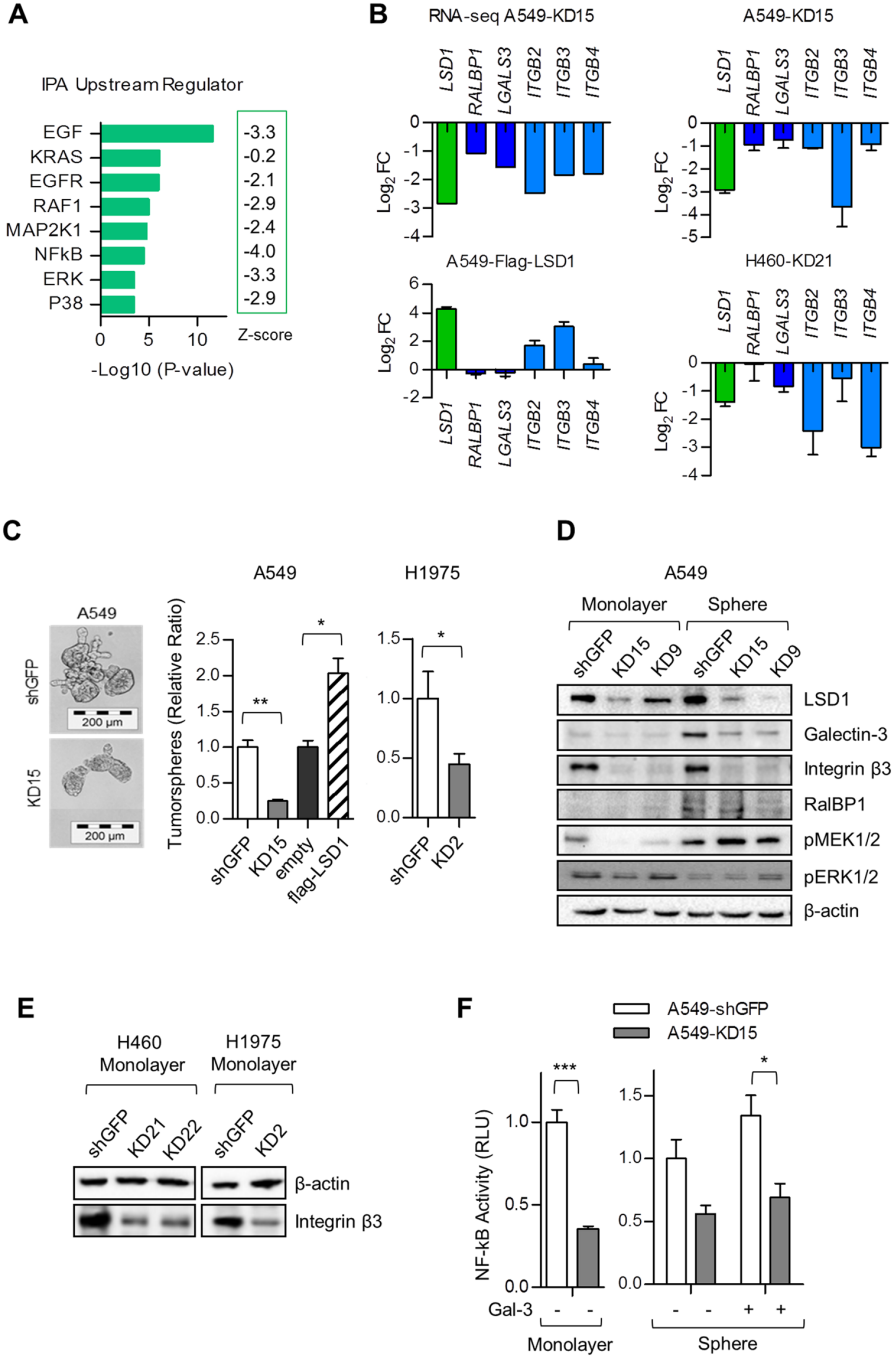
LSD1 contributes to self-renewal by modulating integrin β3-KRAS-NF-κB pathway. (**A)** The predicted upstream regulators of the LSD1 knockdown. Significant overlap (Log_10_ p-values) between differentially expressed genes in A549 RNA-seq and known target genes regulated by each transcriptional regulator (Upstream Regulator) was assessed by Ingenuity Upstream Regulators analysis. The activation state (activation z-score) of an upstream regulator is determined by a literature-derived regulation direction associated with the relationship from the regulator to the differentially regulated genes in RNA-seq datasets. The negative activation z-scores infer the “inhibiting” regulation direction of described Upstream Regulators. (**B**) Influence of the LSD1 knockdown or overexpression on the expression of members of the non-canonical integrin β3 pathway. RNA-seq data of LSD1 stably silenced A549 KD15 vs. A549 shGFP cells (RNA-seq A549) demonstrate downregulation of the *RALBP1, LGALS3, ITGB2, ITGB3, and ITGB4*. LSD1 mediated regulation of genes, involved in integrin β3 signaling, was confirmed by qRT-PCR comparing transcript levels of A549 KD15 vs. A549 shGFP cells (A549-KD15), the A549 LSD1 overexpressing cells vs. the mock control (A549-Flag-LSD1) and the LSD1 knockdown H460 cells vs. shGFP H460 cells (H460-KD21). Changes in transcript levels were calculated by ΔΔCt values and expressed as Log_2_ fold change (FC). **(C)** Phase contrast microscope images of self-renewal tumorspheres of A549 expressing non-silencing GFP shRNA or specific LSD1 shRNA (left panel). Effect of LSD1 on tumorsphere formation in A549 and H1975 cells overexpressing or lacking LSD1 measured by quantifying the number of primary tumorspheres (right panel). The bar graph represents the mean ± SEM for n = 3 independent experiments. Scale bar, 200 µm. (**D**) Effect of LSD1 knockdown on galectin-3-integrin β3-RalBP1 pathway in A549 cells. Immunoblot analysis of whole cell lysates from monolayer and 3D tumorsphere culture was done. Data are representative of at least two independent experiments. (**E**) Effect of stable LSD1 knockdown on integrin β3 protein expression in H460 (KD21, KD22) and H1975 (KD2) cells in comparison to the corresponding shGFP control cell line. Immunoblot analysis of whole cell lysates was done. Data are representative of at least three independent experiments. (**F**) Effect of LSD1 knockdown on NF-κB transcriptional activity measured by luciferase assay. Data are expressed in relative luciferase units (RLU). The bar graph represents the mean ± SEM for n = 3. The *P*-value was estimated using Student’s t-test in D and F. *P < 0.05, **P < 0.01, ***P < 0.001.

IPA Upstream Regulator analysis suggested that transcriptional target genes of the EGF/EGFR and KRAS signaling pathway were negatively regulated in LSD1 silenced A549 cells (Fig. 3A). In particular, signaling components related to the integrin β3-KRAS-NF-κB signaling axis, previously described by Seguin *et al*.^7^, were affected. Thus, galectin-3 (*LGALS3*), RalA binding protein (*RALBP1*) and integrin β3 (*ITGB3*) were significantly downregulated by LSD1 knockdown of A549 cells (Fig. 3B). In order to validate downregulation of members involved in the non-canonical integrin β3 signaling, we generated LSD1 overexpressing A549 cells by stable transfection with a Flag-LSD1 construct (Supplementary Fig. S1B) and analyzed the expression profiles by quantitative RT-PCR. Whereas A549 LSD1 knockdown cells showed downregulation of the integrin β3 signaling member genes, we observed an upregulation of these genes in A549 cells stably expressing the flag-LSD1 construct (Fig. 3B). The effect of LSD1 on gene expression of galectin-3, *RALBP1*, and integrin β3 could be further confirmed on H460 NSCLC LSD1-knockdown cells (Fig. 3B).

Previous work showed that the non-canonical integrin β3 pathway is involved in stem cell like properties and erlotinib resistance^7^. In order to test self-renewal capacity, we analyzed tumor cell growth in 3-dimension (3D) cell culture conditions. Knockdown of LSD1 impaired tumorsphere formation, whereas stable overexpression of LSD1 clearly enhanced 3D colony formation (Fig. 3C). Since it has been shown that an integrin β3 positive stem-like tumor cell population is selectively resistant to receptor tyrosine kinase inhibitors such as erlotinib, we exposed A549 cells to erlotinib^7^. In agreement with these previous findings, we observed that knockdown of LSD1 sensitized A549 cells to erlotinib treatment, decreasing the 3D cell growth capability (Supplementary Fig. S1C).

Subsequently, signaling components related to the integrin β3-KRAS-NFκB signaling axis were analyzed in LSD1 silenced A549 monolayers and 3D tumorspheres, respectively (Fig. 3D). The integrin β3 protein level was significantly diminished upon LSD1 knockdown in A549 cells as well as in other integrin β3 positive cells such as H460 or H1975 (Fig. 3E). Galectin-3 and RalBP1 of which basal levels were higher in A549 spheroids than in the monolayer culture, were prominently downregulated in the sphere condition of LSD1 silenced A549 cells (Fig. 3D). Knockdown of LSD1, however, failed to abrogate KRAS downstream MEK1/2 signaling in the sphere cultures, suggesting that KRAS-MEK1/2 signaling is not essential for LSD1-mediated 3D colony formation (Fig. 3D).

Next, we investigated whether LSD1 affects the NF-κB mediated transcriptional response using a luciferase reporter expression system containing NF-κB responsive elements. Knockdown of LSD1 significantly decreased basal NF-κB transcriptional activity (Fig. 3F). In addition, transcription of NF-κB target genes was greatly inhibited upon LSD1 knockdown (Supplementary Fig. S1D). Galectin-3, the non-canonical ligand of integrin β3, has been reported to facilitate the integrin β3-KRAS interaction leading to NF-κB activation^7^. Addition of recombinant galectin-3 slightly enhanced NF-κB activity, however, did not recover the LSD1 knockdown-mediated blockage of NF-κB pathway possibly due to the strong downregulation of integrin β3 in LSD1 silenced cells (Fig. 3F).

### The LSD1-integrin β3 axis in NSCLC-AC

**Figure 4:**
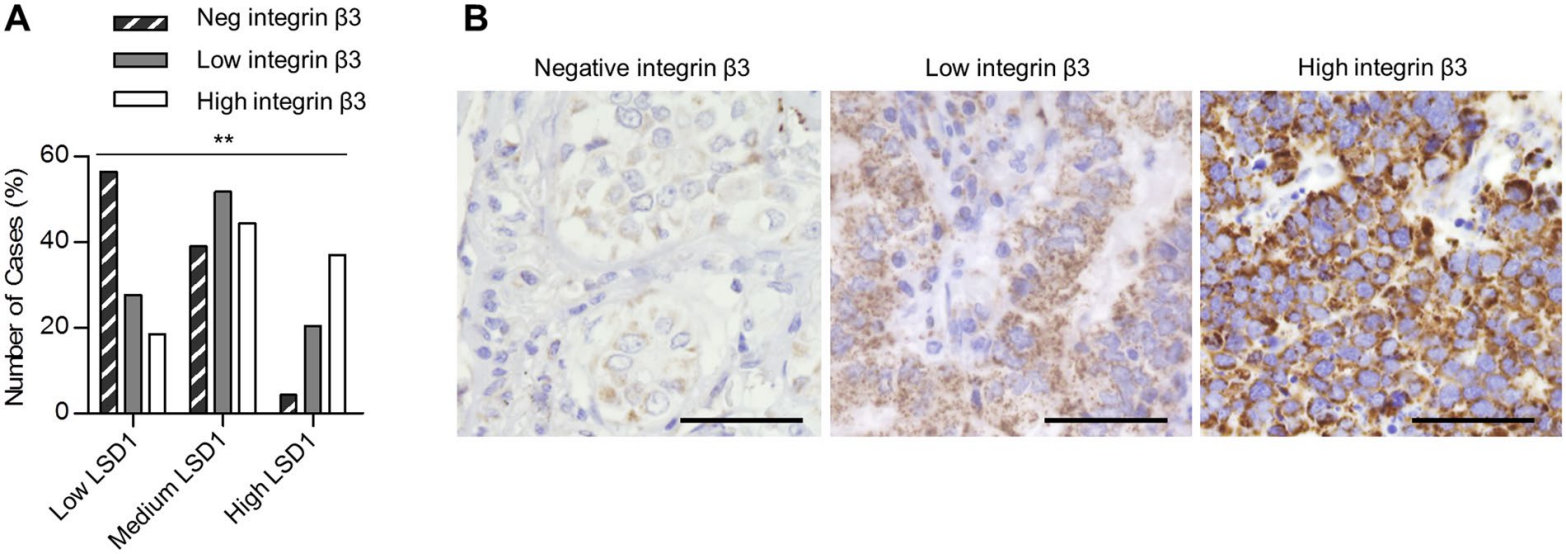
The LSD1-integrin β3 axis in NSCLC adenocarcinoma. (**A**) Immunohistostaining of integrin β3 on 182 lung adenocarcinomas was classified into three integrin β3 expression groups (negative, low, and high). Chi-Square tests were used to calculate the statistical significance for linear-by-linear association. *p* = 0.001. Original data can be found in Supplementary Table S2. (**B**) Representative images of negative, moderate and strong immunohistochemical stainings of integrin β3. Scale bars, 50 µm.

After having demonstrated the impact of LSD1 on integrin β3 expression, we studied whether LSD1 expression might influence the integrin β3 level in clinical specimens from lung AC patients. Indeed, the association between LSD1 and integrin β3 was shown to be highly significant. Negative integrin β3 staining was observed prominently in low LSD1 expression group but very rarely in high LSD1 expression group (Fig. 4A and B), which supports a functional link between LSD1 and integrin β3 expression *in vivo*.

### LSD1 knockdown impacts differentiation of A549 cells

**Figure 5:**
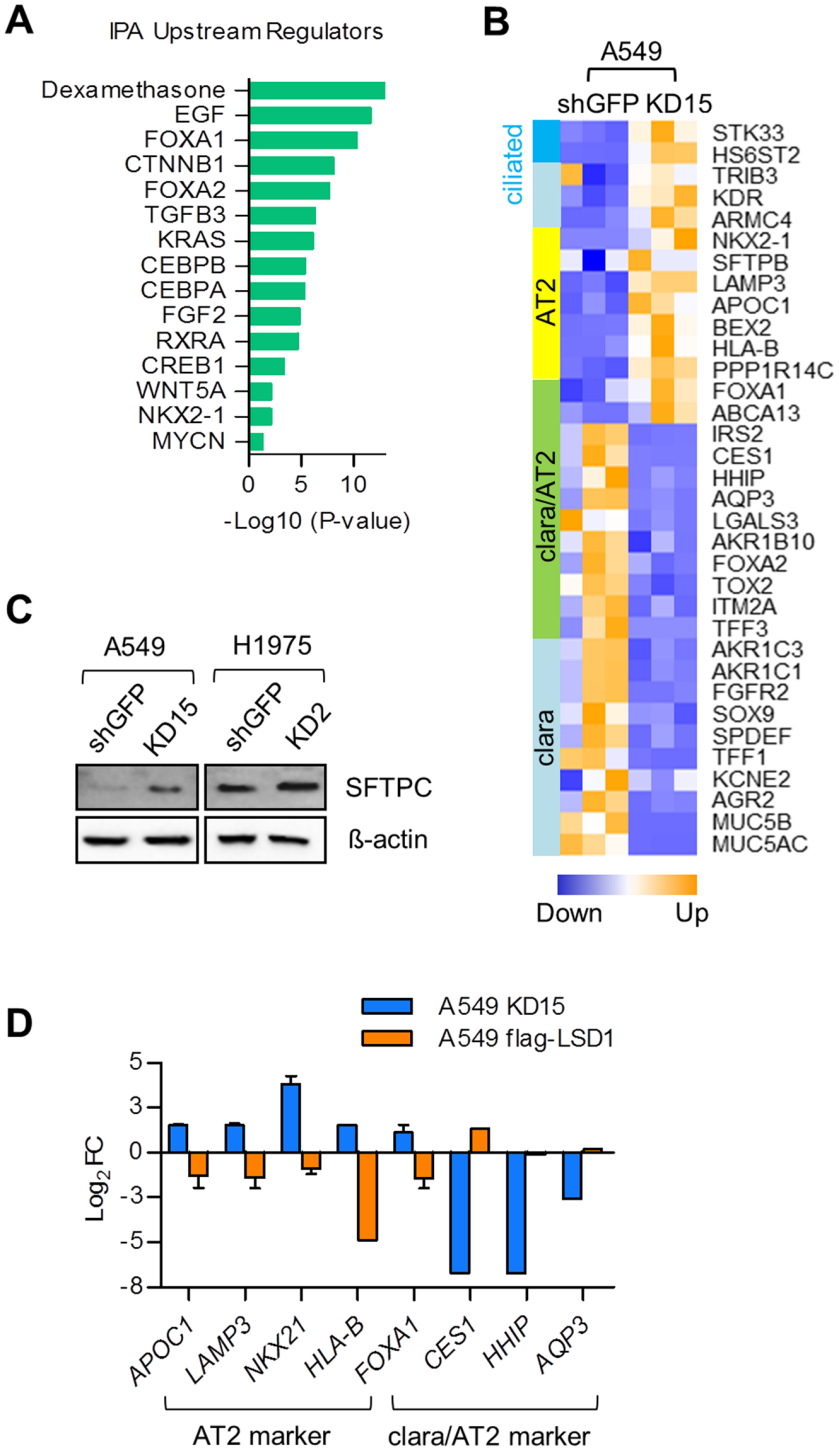
LSD1 knockdown impacts differentiation of A549 cells. (**A**) The bar graph showing the *Upstream Regulators* involved in lung development affected by LSD1 knockdown in A549 cells assessed by IPA. (**B**) A heatmap showing differential gene expression of known markers for AT2, clara and ciliated clara cells measured by RNA-seq. Upregulation of gene upon LSD1 knockdown is indicated in orange and downregulation of gene is indicated in blue. (**C**) Effect of LSD1 knockdown on SFTPC expression determined by western blot. (**D**) The bar graph showing the change in mRNA expression level of AT2 and clara cell marker genes upon LSD1 knockdown or overexpression in A549 cells determined by quantitative real-time PCR. Log_2_(A549 KD15/A549 shGFP) in blue, Log_2_(A549 flag-LSD1/A549 empty) in orange.

As detailed above, LSD1 knockdown induced significant changes in gene expression profiles of key mediators of cell morphogenesis in differentiation (Fig. 2C and Supplementary Fig. S2A). Further, LSD1 conferred tumor cells a stem-like phenotype by modulating the integrin β3-KRAS-NF-κB pathway. We thus hypothesize that LSD1 might be a critical factor affecting the differentiation state of lung AC A549 cells. Targets of the lung lineage transcription factors FOXA1/2 and NKX2-1 were highly affected by LSD1 silencing (Fig. 5A)^24,25^. In addition, the transcriptional responsive genes of dexamethasone, a synthetic glucocorticoid and C/EBPα/β, both of which are involved in late alveolar maturation, were greatly regulated by LSD1 knockdown (Fig. 5A)^24^.

Furthermore, a survey of lung epithelial marker genes revealed that many hallmarks of alveolar type 2 (AT2) and bronchial clara cell markers were altered reflecting a change in cell differentiation state upon LSD1 knockdown (Fig. 5B). A549 cells primarily originated from AT2 cells^26^, appear to have distorted molecular signatures such as the loss of AT2 marker genes and the aberrant gain of clara cell marker genes. The expression of the AT2 cell marker genes, e.g. *NKX2-1* and *SFTPB* is silenced in A549 cells indicating that the transformed AT2 cells lost their cell identity and are not capable of producing surfactant proteins like SFTPC (Fig. 5B,C)^26^. Inhibition of LSD1 partially reactivated AT2 cell marker gene expression while on the other hand it decreased expression of genes responsible for the clara cell phenotype (Fig. 5B,C and Supplementary Fig. S2B). Finally, some of AT2 and clara cell marker genes were suggested to be directly targeted by LSD1, as gene regulation upon LSD1 knockdown was reversed by overexpression of LSD1 in A549 (Fig. 5D).

## Discussion

In our study, we found that LSD1 expression level varied considerably among the different subtypes of lung cancer. RNA-seq analysis of 198 lung cancer specimens showed highest LSD1 mRNA levels in SCLC, which might explain the marked effect of the LSD1 inhibitor GSK2879552 in SCLC cell lines^18^. In comparison to SCLC, AC presented with lower LSD1 mRNA levels. However, analysis of LSD1 expression in 182 AC specimens showed that high LSD1 expression correlated with enhanced lung tumor malignancy. Strong LSD1 expression co-occurred with higher tumor grade and lymphatic invasion, suggesting a crucial role of LSD1 in cellular dedifferentiation and metastasis in lung AC.

Kong *et al*. reported that NSCLCs carrying KRAS mutations have a lower LSD1 expression than those harboring EGFR mutations^17^. In our study we observed only a slightly reduced LSD1 expression in AC with an activating KRAS mutation compared to AC, which did not harbor a KRAS mutation. For our further studies we established stable LSD1 knockdown cells from NSCLC cell types. Although LSD1 knockdown-mediated influence seems to have less effect on the cellular proliferation in early passages, cell growth was inhibited in late passages. In A549 and H1975 KD cells, we show a pronounced impact on the oncogenic transformational capacities such as invasion and self-renewal by LSD1 knockdown. Accordingly, the constitutive activation of MEK1/2-ERK pathways, known to be crucial for cell proliferation, was not blocked by LSD1 knockdown in A549 cells. Instead, galectin-3 and integrin β3 expression was severely downregulated by LSD1 silencing. This suggests that LSD1 is rather implicated in cancer stem cell function by regulating galectin-3-integrin β3-KRAS signaling^7^. Interestingly, galectin-3 has been identified as the marker gene for alveolar type 1 (AT1) cells^27^ and damage in AT1 cells can induce the activation of KRAS signal axis invoking a stemness phenotype in lung alveolar progenitor cells^28^. Similarly to galectin-3, galectin-1 has recently been identified to promote lung cancer metastasis by potentiating integrin α6β4 and Notch1/Jagged2 signaling^29^. This indicates an important role of galectins as signal modulators that activate various oncogenic signal pathways during tumor progression.

Whereas the contribution of LSD1 to proliferation is not clear in KRAS mutated AC, there is accumulating evidence that LSD1 is significantly associated with cell invasion, migration and metastasis in NSCLC^16,17^. Previous studies showed that LSD1 is required for upregulation of epithelial-mesenchymal transition (EMT) markers including twist family bHLH transcription factor 1 (TWIST1), E-cadherin and N-cadherin and downregulation of metallopeptidase inhibitor TIMP3, thereby contributing to cellular invasion and migration^16,17^. Here, we performed a comprehensive expression profiling using RNA-seq showing that LSD1 knockdown in A549 cells induced a global transcriptome change. EMT signaling pathways and EMT markers such as Zinc finger E-box binding homeobox 2 (*ZEB2*), KIT proto-oncogene receptor tyrosine kinase (*KIT*), and cadherins (*CDH*s) were differentially regulated in agreement with the previous studies (Fig. 2C)^16,17^. Furthermore, pathways associated with invasive phenotypes such as endocytosis, RhoA and integrin signaling were affected by LSD1 knockdown.

Most importantly, LSD1 knockdown interfered with integrin β3-KRAS signaling axis by a pronounced integrin β3 repression. Recently, a number of integrin subunits, particularly β3 and β4 have been highlighted as markers and functional regulators of normal adult stem and progenitor cells, as well as cancer stem cells^30^. Integrin β3 is enriched in mammary luminal progenitors, whereas differentiated luminal cells lack integrin β3^31^. In breast and pancreatic tumors, expression of integrin αvβ3 is necessary to drive tumor cell anchorage-independence and metastasis by recruiting and activating Src kinase^32^. In the Kras^G12D^;Trp53^fl/fl^ lung cancer model, integrin β4 has been characterized as a new cancer stem cell marker involved in self-renewal, tumor propagation and cisplatin resistance^33^. Integrin α6β4 positive cell subpopulation from mouse lung could expand clonally as progenitors but also differentiate into mature airway and alveolar cells^34,35^.

In light of this data, blocking LSD1 appears to be critical to impair integrin β3-KRAS mediated stemness along with invasive phenotype by downregulating integrin β3. In agreement, LSD1 expression was higher in poorly differentiated and metastasized adenocarcinomas and correlated with the expression of integrin β3 *in vivo*. In agreement to the previous studies of Seguin *et al*.^7^, the integrin β3-KRAS signal axis is also important for the invasive and self-renewal ability of H1975 cells due to their activation by the EGFR/KRAS pathway. Since we show that LSD1 affects integrin β3 expression, as well as self-renewal and invasion capacities of H1975 cells, the LSD1 influence on this non-canonical integrin β3 signaling seems to be also valid for the EGFR mutant cell context. While potentiating stemness, LSD1 suppresses pro-differentiation processes as indicated by the altered expression of genes involved in cell morphogenesis in differentiation and late alveolar maturation upon LSD1 knockdown. A549 cells which are derived from AT2 cells, have distorted molecular signatures such as the aberrant gain of airway cell marker genes. This suggests that transformed A549 AC cells mimic a cell type with partially bronchioalveolar progenitor-like gene signature expressing either bronchial or alveolar marker gene expression^4^. LSD1 inhibition could partially reactivate expression of silenced AT2 marker genes, whereas decrease expression of bronchial marker genes, altering cell differentiation state in A549 cells, a mechanism similar to the observed pro-differentiation effect in SCLC and leukemia upon loss of LSD1^15,18^.

Taken together, our data show that LSD1 confers lung adenocarcinoma cells with invasive and dedifferentiated attributes by modulating a non-canonical integrin β3-KRAS signaling pathway. Pharmacological LSD1 inhibition in combination with anti-proliferative cancer drugs might be a rational strategy for the treatment of lung adenocarcinoma to suppress tumor proliferation while preventing metastatic phenotype.

## Methods

### Patients’ tissue specimens and TMA preparation

Lung adenocarcinoma tissue specimens were collected from the archive of the Institute of Pathology, University Hospital Cologne, Germany. Formalin-fixed, paraffin-embedded tissues of 182 lung adenocarcinomas were used to prepare tissue microarrays as previously described^36^. Clinicopathological parameters were obtained from patient records. In addition, the KRAS and EGFR mutation status of tumor specimens was determined by routine diagnostic processing using next generation sequencing as described previously^37^. The characteristics of the cohort are summarized in Table 1. The study was approved by the ethics committee (KEK Nr. 200/2014) and informed consent was obtained from all patients. All methods were performed in accordance with the relevant guidelines and regulations of the institution.

### Immunohistochemistry

Immunohistochemical staining was done on 4 μM sections of paraffin-embedded tumors from TMA as described previously^13,36^ using the primary α-LSD1 antibodies (1:250) (Abcam, Cambridge, UK) and α-integrin β3, (1:500) (Abcam). Immunohistochemistry was then performed on an automated staining system (Lab Vision Autostainer 480 S, Thermo Scientific, Waltham, USA) according to standardized protocol of the supplier. Nuclear immunostaining results for LSD1 were scored between 1 and 3 (1 = low, 2 = medium, 3 = high) by two experienced pathologists (SS, LO) (Fig. 1). Integrin β3 staining intensity was scored on a scale of 0–3 (0 = negative, 1, 2 = low, 3 = high).

### Cell culture

A549, H460, PC9, H1975, HCC827, H3122 and H2228 cells were cultivated in DMEM or RPMI medium supplemented with 10% fetal calf serum, L-glutamine and antibiotics (Invitrogen, Carlsbad, USA). PSAE and NuLi-1 cells were purchased from ATCC and cultivated according to the protocol of ATCC.

### Genetic knockdown and LSD1 overexpression

A549, H460, H1975, lung cancer cell lines were transfected with GFP shRNA control (shGFP) or LSD1 shRNA using a pSUPER retroviral system. Using the puromycin resistance, cells were screened for recombination and from single cells monoclonal cell populations were established. After checking the LSD1 knockdown, A549, H460, and H1975 monoclonal cell cultures expressing shLSD1 or shGFP were used only in passages 5 to 8 to ensure stability of the cell culture system. Therefore, not all KD clones could be used in the study, but from A549 cell line the KD clones 9 and 15, from the H460 the clones KD21 and 22, and from H1975 clone KD2 were applied to LSD1 functional analysis.

Furthermore, for LSD1 overexpression, cells were transfected with empty vector control or flag-LSD1 expression vector using a lentiviral system. Gene silencing and overexpression was confirmed by immunoblot analysis. The following plasmids were used: pSUPER.retro.shGFP.puro (a kind gift from Michael Hölzel, University Hospital Bonn, Germany) and pSUPER.retro.shLSD1.puro, pSIN-flag-LSD1-puro and pSIN-empty-puro. pSIN plasmids were modified from pSIN-EF2-Nanog-puro vector (Addgene plasmid #16578) and pCMX-flag-LSD1 (a kind gift from Roland Schüle, University Freiburg Medical Center, Germany)^11^. The shRNA sequence for LSD1 (AAGGAAAGCTAGAAGAAAA) was cloned to pSUPER.retro.puro plasmid (OligoEngine, Seattle, USA) according to manufacturer’s protocol.

### RNA sequencing and data analysis

Expression levels of LSD1 were determined by referring to the RNA-seq data of lung ACs (n = 40)^19,20^, SQs (n = 9)^19^, pulmonary carcinoids (n = 69)^21^, and SCLC (n = 81)^3^. Expression values were determined with Cufflinks and represented as fragments per kilobase of exon per million fragments mapped (FPKM).

For transcriptome sequencing, total RNA from three biological replicates of each A549 shLSD1 KD15 cells and A549 shGFP cells was isolated using the RNeasyMini kit (Qiagen Inc, Hilden, Germany). The Illumina TruSeq RNA Sample Prep Kit (Cat#FC-122-1001, Illumina, San Diego, USA) was used with 1 µg of total RNA for the construction of sequencing libraries. RNA libraries were prepared for sequencing using standard Illumina protocols. Fastq files were obtained after demultiplexing using Illuminas CASAVA v1.8.2 pipeline with default parameters. Reads were mapped against the human genome GRCh37/hg19 using BWA v0.5.9-r16 with default parameters. Exon read coverages were obtained with coverageBed (Bedtools-v2.17.0) using exon coordinates from Ensembl database v73. Subsequent analyses were done using the edgeR package in R^38^. Expression changes were calculated as log2 ratios and *p*-values were adjusted for multiple testing by controlling the Benjamini-Hochberg false discovery rate at 5%. Ingenuity (Qiagen Inc) pathway analysis was used to find enriched canonical pathways, and Upstream Regulators. Furthermore, the database for annotation, visualization and integrated discovery (DAVID) was used to identify the enriched gene ontology terms^39^.

### Proliferation and transwell invasion assay

Cell proliferation assays were carried out as described previously^13^. Briefly, cells were seeded at a density of 2,500 cells per well in 96 well microplates and cultured in standard medium. Viable cell numbers were determined 0, 1, 2, 3 and 6 days after cell plating using CellTiter 96 Aqueous Cell Proliferation assay kit (Promega, Madison, USA). For invasion assay, Boyden Chambers (Cell Biolabs, San Diego, USA) were layered with 2 mg/ml extracellular matrix gel from Engelbreth-Holm-Swarm mouse sarcoma (Sigma-Aldrich, St. Louis, USA) overnight at 37 °C. Cells were seeded at a density of 100,000 per well. Wells were then filled with standard medium. After 48 h, Boyden chambers were washed twice with PBS, followed with fixation of the cells using 4% formaldehyde (Sigma Aldrich). Subsequently, cells were stained with 1% Crystal Violet (Sigma Aldrich), photographed and counted.

### Tumor-sphere cell culture

Tumorsphere assays were carried out as described by Seguin *et al*.^7^. Single cells were seeded on ultralow attachment plates (Corning, New York, USA) at a concentration of 2,000 cells/ml in DMEM/F12 medium supplemented with insulin–transferrin–selenium, 50 ng/ml EGF and 20 ng/ml bFGF (Invitrogen). Tumorspheres larger than 50 μm in diameter were counted 7 days after seeding. To analyze erotinib resistance in A549 cells, cells were seeded at a density of 3,000 cells per well into ultralow adherent 96-well plates (Corning). The cells were then treated with 1 µL DMSO and erlotinib (1 µM–20 µM) (Sigma-Aldrich) for three days. Viable cell numbers were determined using the CellTiter 96 Aqueous Cell Proliferation assay kit (Promega).

### Luciferase assay

Cells were transfected with NF-kB luciferase reporter vector (pNF-kB-luc, Clontech, Mountain View, USA) together with pRL Renilla luciferase control reporter vector (pRL-null, Promega) using lipofectamine (Invitrogen) according to the recommendation of the manufacturer. After 24 h incubation, cells were transferred into the ultra-low adherent well plates (Corning). Next day, cells were treated with 2.5 µg/ml human recombinant galectin-3 (PepproTech, Rocky Hill, USA) for 4 hours. Luciferase assays were then carried out using the Dual Luciferase Reporter assay kit (Promega) according to the manufacturer’s protocol. Each assay consisted of three or four replicates and each experiment was repeated at least three times. Data were presented as relative luciferase units (RLU) normalized to the Renilla luciferase signal.

### Western blot analysis

Protein lysates were extracted from cells using the cell lysis buffer (Cell Signaling, Danvers, USA) and blotted as described in Lim *et al*.^13^. The membranes were incubated overnight using the following antibodies and dilutions: α-LSD1 (Abcam) 1:1000; α-Galectin-3 (Leica, Wetzlar, Germany) 1:1000; β-actin (Sigma-Aldrich) 1:5000; α-Integrin β3 (Abcam) 1:1000; α-RalBP-1 and α-SFTPC (Santa-Cruz, Dallas, USA) 1:200; pMEK1/2 and pERK1/2 (Cell Signaling) 1:1000.

### Quantitative real time-PCR

Complementary DNA was synthesized using the AB Reverse Transcription kit using random primers (Applied Biosystems, Foster City, USA) and quantitative PCR was carried out on real-time PCR machine (BioRad, Hercules, USA) with SYBR reagent (Promega). Expression values were normalized to the mean of HPRT1 and the 2^*−ΔΔCT*^ method was applied to calculate relative gene expression levels. A list of primers used for qRT-PCR validation is available in Supplementary Table S4.

### Statistical analysis

Statistical analysis for immunohistochemistry was performed using SPSS 17.0 program (SPSS) and Chi-Square tests were used to calculate the statistical significance for linear-by-linear association. Mann-Whitney U test or Student’s T-tests were used to calculate statistical significance using GraphPad Prism software. A P-value less than 0.05 was considered to be significant. *P < 0.05, **P < 0.01, ***P < 0.001.

### Accession numbers

The RNA-seq data of A549 cells has been deposited in Gene Expression Omnibus under accession code GSE86874.

## Electronic supplementary material

Supplementary Information

## References

[1] Klein CA (2009). Parallel progression of primary tumours and metastases. Nat. Rev. Cancer.

[2] A genomics-based classification of human lung tumors. *Sci. Transl. Med*. **5**, 209ra153 (201p3). 10.1126/scitranslmed.3006802 PMC4006630 24174329

[3] George J (2015). Comprehensive genomic profiles of small cell lung cancer. Nature.

[4] Leeman KT, Fillmore CM, Kim CF (2014). Lung stem and progenitor cells in tissue homeostasis and disease. Curr. Top. Dev. Biol..

[5] Adhikari AS, Agarwal N, Iwakuma T (2011). Metastatic potential of tumor-initiating cells in solid tumors. Front Biosci. (Landmark Ed).

[6] Desgrosellier JS, Cheresh DA (2010). Integrins in cancer: biological implications and therapeutic opportunities. Nat. Rev. Cancer.

[7] Seguin L (2014). An integrin beta(3)-KRAS-RalB complex drives tumour stemness and resistance to EGFR inhibition. Nat. Cell Biol..

[8] Baylin SB, Jones PA (2011). A decade of exploring the cancer epigenome - biological and translational implications. Nat. Rev. Cancer.

[9] Barlesi F (2007). Global histone modifications predict prognosis of resected non small-cell lung cancer. J. Clin. Oncol..

[10] Shi Y (2004). Histone demethylation mediated by the nuclear amine oxidase homolog LSD1. Cell.

[11] Metzger E (2005). LSD1 demethylates repressive histone marks to promote androgen-receptor-dependent transcription. Nature.

[12] Kahl P (2006). Androgen receptor coactivators lysine-specific histone demethylase 1 and four and a half LIM domain protein 2 predict risk of prostate cancer recurrence. Cancer Res..

[13] Lim S (2010). Lysine-specific demethylase 1 (LSD1) is highly expressed in ER-negative breast cancers and a biomarker predicting aggressive biology. Carcinogenesis.

[14] Schulte JH (2009). Lysine-specific demethylase 1 is strongly expressed in poorly differentiated neuroblastoma: implications for therapy. Cancer Res..

[15] Harris WJ (2012). The histone demethylase KDM1A sustains the oncogenic potential of MLL-AF9 leukemia stem cells. Cancer Cell.

[16] Lv T (2012). Over-expression of LSD1 promotes proliferation, migration and invasion in non-small cell lung cancer. PLoS One.

[17] Kong L (2016). KDM1A promotes tumor cell invasion by silencing TIMP3 in non-small cell lung cancer cells. Oncotarget.

[18] Mohammad HP (2015). A DNA Hypomethylation Signature Predicts Antitumor Activity of LSD1 Inhibitors in SCLC. Cancer Cell.

[19] Fernandez-Cuesta L (2015). Identification of novel fusion genes in lung cancer using breakpoint assembly of transcriptome sequencing data. Genome Biol..

[20] Fernandez-Cuesta L (2014). CD74-NRG1 fusions in lung adenocarcinoma. Cancer Discov..

[21] Fernandez-Cuesta L (2014). Frequent mutations in chromatin-remodelling genes in pulmonary carcinoids. Nat. Commun..

[22] Battafarano RJ (2005). Large cell neuroendocrine carcinoma: an aggressive form of non-small cell lung cancer. J. Thorac. Cardiovasc. Surg..

[23] Barletta JA, Yeap BY, Chirieac LR (2010). Prognostic significance of grading in lung adenocarcinoma. Cancer.

[24] Maeda Y, Dave V, Whitsett JA (2007). Transcriptional control of lung morphogenesis. Physiol. Rev..

[25] Warburton D (2010). Lung organogenesis. Curr. Top. Dev. Biol..

[26] Watanabe H (2013). Integrated cistromic and expression analysis of amplified NKX2-1 in lung adenocarcinoma identifies LMO3 as a functional transcriptional target. Genes Dev..

[27] Treutlein B (2014). Reconstructing lineage hierarchies of the distal lung epithelium using single-cell RNA-seq. Nature.

[28] Desai TJ, Brownfield DG, Krasnow MA (2014). Alveolar progenitor and stem cells in lung development, renewal and cancer. Nature.

[29] Hsu YL (2013). Galectin-1 promotes lung cancer tumor metastasis by potentiating integrin alpha6beta4 and Notch1/Jagged2 signaling pathway. Carcinogenesis.

[30] Seguin L, Desgrosellier JS, Weis SM, Cheresh DA (2015). Integrins and cancer: regulators of cancer stemness, metastasis, and drug resistance. Trends. Cell Biol..

[31] Asselin-Labat ML (2007). Gata-3 is an essential regulator of mammary-gland morphogenesis and luminal-cell differentiation. Nat. Cell. Biol..

[32] Desgrosellier JS (2009). An integrin alpha(v)beta(3)-c-Src oncogenic unit promotes anchorage-independence and tumor progression. Nat. Med..

[33] Zheng Y (2013). A rare population of CD24(+)ITGB4(+)Notch(hi) cells drives tumor propagation in NSCLC and requires Notch3 for self-renewal. Cancer Cell.

[34] Chapman HA (2011). Integrin alpha6beta4 identifies an adult distal lung epithelial population with regenerative potential in mice. J. Clin. Invest..

[35] McQualter JL, Yuen K, Williams B, Bertoncello I (2010). Evidence of an epithelial stem/progenitor cell hierarchy in the adult mouse lung. Proc. Natl. Acad. Sci. USA.

[36] Schultheis AM (2015). PD-L1 expression in small cell neuroendocrine carcinomas. Eur. J. Cancer.

[37] Konig K (2015). Implementation of Amplicon Parallel Sequencing Leads to Improvement of Diagnosis and Therapy of Lung Cancer Patients. J. Thorac. Oncol..

[38] Robinson MD, McCarthy DJ, Smyth GK (2010). edgeR: a Bioconductor package for differential expression analysis of digital gene expression data. Bioinformatics.

[39] Huang da W, Sherman BT, Lempicki RA (2009). Systematic and integrative analysis of large gene lists using DAVID bioinformatics resources. Nat. Protoc..

