# Author Correction: LSD1 modulates the non-canonical integrin β3 signaling pathway in non-small cell lung carcinoma cells

**DOI:** 10.1038/s41598-018-31451-0

**Published:** 2018-11-01

**Authors:** So-Young Lim, Iris Macheleidt, Priya Dalvi, Stephan C. Schäfer, Martin Kerick, Luka Ozretić, Sandra Ortiz-Cuaran, Julie George, Sabine Merkelbach-Bruse, Jürgen Wolf, Bernd Timmermann, Roman K. Thomas, Michal R. Schweiger, Reinhard Buettner, Margarete Odenthal

**Affiliations:** 1 Institute of Pathology, University Hospital of Cologne, 50931 Cologne, Germany; 2 The Center for Molecular Medicine Cologne (CMMC), 50931 Cologne, Germany; 3 Center of Integrative Oncology, University Clinic of Cologne and Bonn, 50937 Cologne, Germany; 4 Functional Epigenomics, University of Cologne, 50931 Cologne, Germany; 5 Department of Translational Genomics, Medical Faculty, University of Cologne, 50931 Cologne, Germany; 6 Centre Léon Bérard, 69008 Lyon, France; 7 Lung Cancer Group Cologne, University Hospital of Cologne, 50937 Cologne, Germany; 8 Clinic for Internal Medicine, University Hospital of Cologne, 50937 Cologne, Germany; 9 Max Planck Institute for Molecular Genetics, 14195 Berlin, Germany; 10 German Cancer Research Center, German Cancer Consortium (DKTK), 69120 Heidelberg, Germany

Correction to: *Scientific Reports* 10.1038/s41598-017-09554-x, published online 31 August 2017

In the original version of this Article, Affiliation 5 was incomplete and incorrectly listed as ‘Department of Translational Genomics, University of Cologne, 50931, Cologne, Germany’ The correct affiliation is listed below:

Department of Translational Genomics, Medical Faculty, University of Cologne, 50931 Cologne, Germany

In addition, the Acknowledgements was incomplete. It now reads:

“This work was supported by Center for Molecular Medicine Cologne (CMMC) to RB and MO and by the German Cancer Aid as part of the Interdisciplinary Oncology Centers of Excellence program to the Center for Integrated Oncology Köln Bonn. In addition, the project was supported by the Federal German Ministry of Science and Education (BMBF) as part of the e-Med program (grant no. 01ZX1303A and 01ZX1603A to RB and RKT) and the German federal state North Rhine Westphalia (NRW) and by the European Union (European Regional Development Fund: Investing in Your Future) as part of the PerMed.NRW initiative (grant 005-1111-0025 to RKT and RB) as well as the EFRE initiative (grant LS-1-1-030 to RKT, RB, MS and MO), by the German Consortium for Translational Cancer Research (DKTK) Joint Funding program (RKT) and a grant from the Volkswagenstiftung (Lichtenberg program) to MS. We highly appreciate the excellent technical assistance of Michael Gentz, Olivia Käsgen and Marion Müller.”

Finally, disclosures were omitted from the Competing Financial Interests statement which now reads:

R.K.T. is a consultant of NEO New Oncology GmbH and received honoraria from AstraZeneca, Bayer, NEO New Oncology GmbH, Boehringer Ingelheim, Clovis Oncology, Daiichi-Sankyo, Eli Lilly, Johnson & Johnson, Merck KGaA, MSD, Puma, Roche and Sanofi.

These errors have now been corrected in the PDF and HTML versions of the Article, and in the accompanying Supplementary Information file.

