# Supplementary material for: LSD1 modulates the non-canonical integrin β3 signaling pathway in non-small cell lung carcinoma cells
Source: Sci Rep. 2017 Aug 31;7:10292. doi: 10.1038/s41598-017-09554-x (PMC5578970; doi:10.1038/s41598-017-09554-x)
Supplement: Supplementary file 1 — Supplementary Information [file 41598_2017_9554_MOESM1_ESM.doc]

**LSD1 modulates the non-canonical integrin β3 signaling pathway in non-small cell lung carcinoma cells**

So-Young Lim^1,2,*#^, Iris Macheleidt^1,2*^, Priya Dalvi^1,2*^, Stephan C. Schäfer^1,3^, Martin Kerick^4^, Luka Ozretić^1^, Sandra Ortiz-Cuaran^3,5,6^, Julie George^3,5^, Sabine Merkelbach-Bruse^1,3,7^, Jürgen Wolf^3,7,8^, Bernd Timmermann^9^, Roman K. Thomas^1,3,5,10^, Michal R. Schweiger^4^, Reinhard Buettner^1,2,3,7^ and Margarete Odenthal^1,2,7^

^1^Institute of Pathology, University Hospital of Cologne, 50931 Cologne, Germany, ^2^The Center for Molecular Medicine Cologne (CMMC), 50931 Cologne, Germany, ^3^Center of Integrative Oncology, University Clinic of Cologne and Bonn, 50937 Cologne, Germany, ^4^Functional Epigenomics, University of Cologne, 50931 Cologne, Germany, ^5^Department of Translational Genomics, Medical Faculty, University of Cologne, 50931 Cologne, Germany, ^6^Centre Léon Bérard, 69008 Lyon, France ^7^Lung Cancer Group Cologne, University Hospital of Cologne, 50937 Cologne, Germany, ^8^Clinic for Internal Medicine, University Hospital of Cologne, 50937 Cologne, Germany ^9^[Max Planck Institute for Molecular Genetics](http://www.molgen.mpg.de/Timmermann), 14195 Berlin, Germany, ^10^German Cancer Research Center, German Cancer Consortium (DKTK), 69120 Heidelberg, Germany

*equally contributed

^#^Corresponding author: so-young.lim@uk-koeln.de

**Supplementary Information**


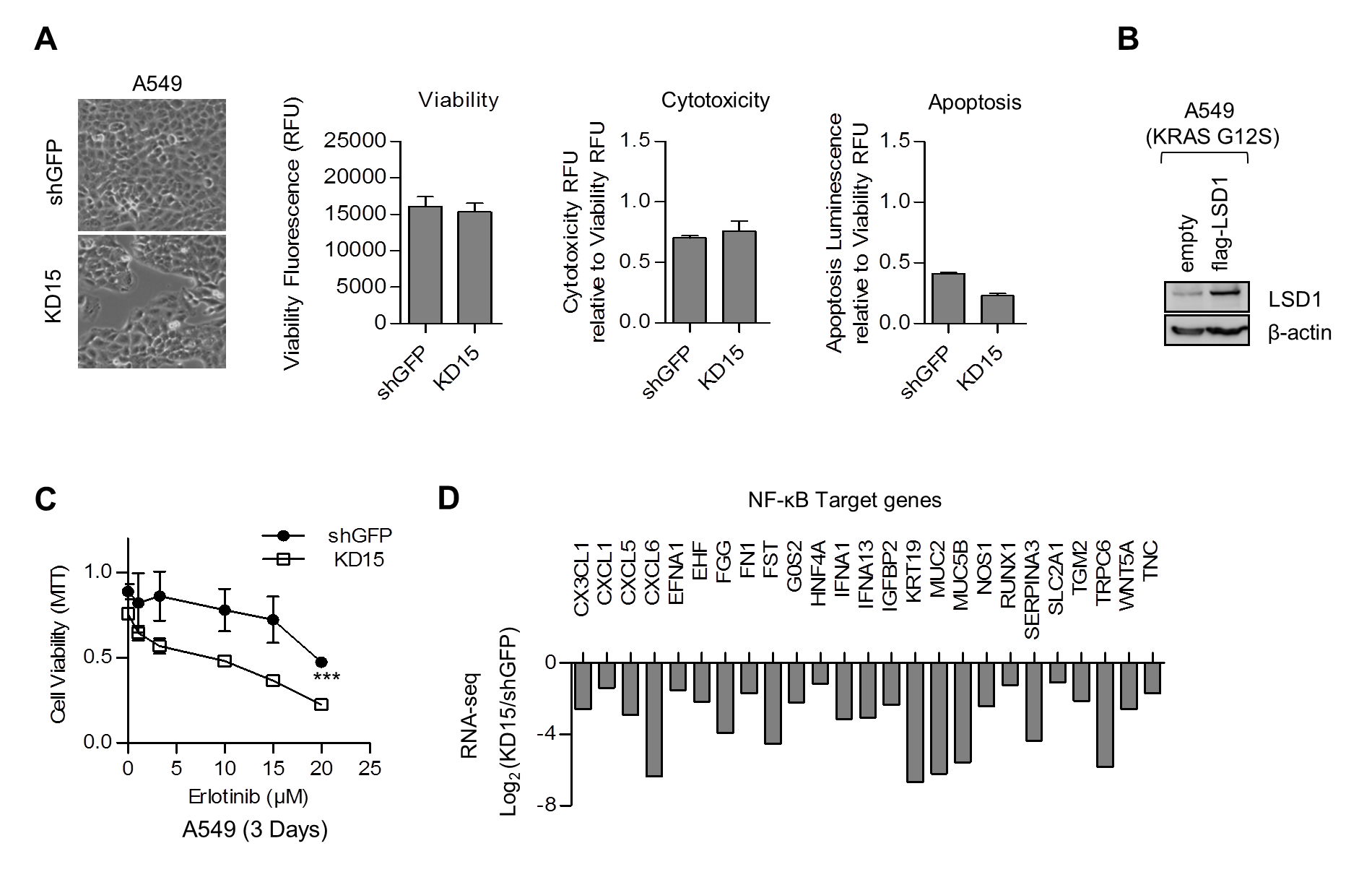


**Fig. S1. LSD1 influence on cell viability, self-renewal, and the integrin β3-KRAS-NF-κB pathway.** Phase contrast microscopy images of A549 GFP and A549 LSD1 KD cells **(A)**. Effect of LSD1 knockdown on cell viability, cytotoxicity and apoptosis was measured by ApoTox-Glo Triplex Assay kit (Promega) **(A)**. Cells were seeded at a density of 2,500 cells per well in 96 well microplates and cultured for 3 days. Neither cytotoxicity nor apoptosis was significantly increased upon knockdown of LSD1 in A549 cells. Immunoblot analysis of A549 flag-LSD1 construct expressing cells, showing higher LSD1 levels as compared to the A549 empty construct control cells **(B).** Effect of LSD1 knockdown on erlotinib resistance was measured by CellTiter 96 AQueous One Solution Cell Proliferation Assay kit **(C)**. A549 cells were grown in 3D cultures and treated with various concentrations of erlotinib for 3 days. Effect of LSD1 knockdown on NF-κB transcriptional target genes as measured by RNA-seq **(D)**. Fold change in mRNA expression levesl of NF-κB target genes upon LSD1 knockdown in A549 were demonstrated as Log_2_-FC (A549 KD15 vs A549 shGFP).


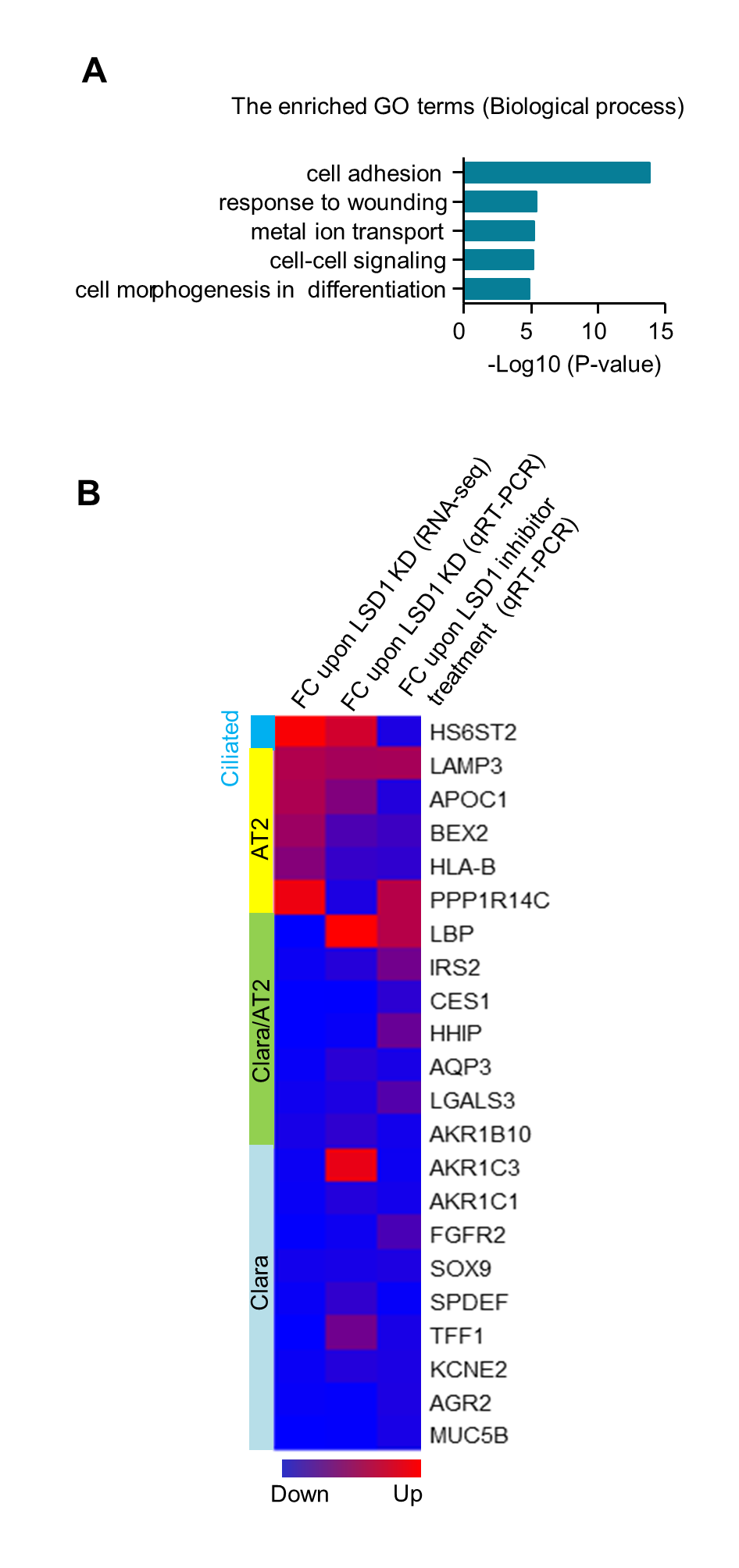


**Fig. S2. Differential gene expression of AT2 and Clara markers upon LSD1 knockdown.** Bar graph showing gene ontology (GO) terms enriched upon LSD1 knockdown in A549 cells assessed by DAVID bioinfomatic resources **(A)**. A heatmap showing differential gene expression of known markers for AT2, clara and ciliated clara cells upon LSD1 knockdown or after 2 µM LSD1 inhibitor HCL-2509 treatment for 3 days in A549 cells measured by qRT-PCR **(B)**. Upregulated genes are indicated in red and downregulated genes are indicated in blue.

**Table S1.** LSD1 mRNA expression in different types of lung tumor as determined by RNA-seq.

| **AD** |  |  | **CA** |  |  | **SQ** |  |  | **SCLC** |  |
| --- | --- | --- | --- | --- | --- | --- | --- | --- | --- | --- |
| sample | KDM1A |  | sample | KDM1A |  | sample | KDM1A |  | sample | KDM1A |
| S00006 | 12 |  | S00016 | 29 |  | S00062 | 78 |  | S00022 | 139 |
| S00021 | 20 |  | S00076 | 24 |  | S00186 | 34 |  | S00035 | 52 |
| S00025 | 12 |  | S00089 | 41 |  | S00473 | 28 |  | S00050 | 64 |
| S00054 | 6 |  | S00094 | 21 |  | S00480 | 6 |  | S00213 | 46 |
| S00059 | 18 |  | S00118 | 22 |  | S01225 | 16 |  | S00356 | 52 |
| S00074 | 22 |  | S00128 | 34 |  | S01265 | 24 |  | S00472 | 47 |
| S00096 | 19 |  | S00515 | 18 |  | S01336 | 19 |  | S00501 | 35 |
| S00167 | 21 |  | S00516 | 17 |  | S01472 | 23 |  | S00825 | 31 |
| S00183 | 22 |  | S00520 | 8 |  | S01751 | 35 |  | S00827 | 42 |
| S00214 | 10 |  | S00716 | 21 |  |  |  |  | S00829 | 82 |
| S00352 | 21 |  | S00858 | 6 |  |  |  |  | S00830 | 42 |
| S00551 | 18 |  | S01060 | 37 |  |  |  |  | S00831 | 53 |
| S00585 | 11 |  | S01103 | 8 |  |  |  |  | S00832 | 76 |
| S00611 | 12 |  | S01202 | 17 |  |  |  |  | S00836 | 47 |
| S00664 | 17 |  | S01493 | 15 |  |  |  |  | S00837 | 47 |
| S00684 | 13 |  | S01501 | 24 |  |  |  |  | S00838 | 38 |
| S00687 | 16 |  | S01502 | 32 |  |  |  |  | S01242 | 98 |
| S00688 | 12 |  | S01504 | 23 |  |  |  |  | S01248 | 70 |
| S00726 | 22 |  | S01510 | 23 |  |  |  |  | S01297 | 64 |
| S00737 | 11 |  | S01513 | 16 |  |  |  |  | S01366 | 35 |
| S00738 | 13 |  | S01515 | 17 |  |  |  |  | S01453 | 65 |
| S00752 | 26 |  | S01519 | 37 |  |  |  |  | S01494 | 58 |
| S00754 | 13 |  | S01520 | 22 |  |  |  |  | S01512 | 23 |
| S00755 | 15 |  | S01521 | 18 |  |  |  |  | S01524 | 32 |
| S01052 | 6 |  | S01526 | 14 |  |  |  |  | S01542 | 73 |
| S01076 | 25 |  | S01528 | 34 |  |  |  |  | S01556 | 93 |
| S01122 | 21 |  | S01529 | 10 |  |  |  |  | S01563 | 72 |
| S01124 | 11 |  | S01531 | 13 |  |  |  |  | S01578 | 34 |
| S01156 | 20 |  | S01532 | 18 |  |  |  |  | S01698 | 65 |
| S01194 | 10 |  | S01536 | 25 |  |  |  |  | S01728 | 53 |
| S01272 | 12 |  | S01537 | 11 |  |  |  |  | S01792 | 69 |
| S01320 | 12 |  | S01538 | 8 |  |  |  |  | S01861 | 39 |
| S01337 | 24 |  | S01539 | 12 |  |  |  |  | S01864 | 62 |
| S01409 | 21 |  | S01540 | 26 |  |  |  |  | S01873 | 60 |
| S01463 | 14 |  | S01541 | 9 |  |  |  |  | S02065 | 122 |
| S01470 | 18 |  | S01543 | 24 |  |  |  |  | S02093 | 27 |
| S01728 | 53 |  | S01545 | 23 |  |  |  |  | S02120 | 51 |
| S01760 | 23 |  | S01546 | 19 |  |  |  |  | S02139 | 55 |
| S01769 | 24 |  | S01567 | 19 |  |  |  |  | S02163 | 111 |
| S01906 | 37 |  | S01572 | 36 |  |  |  |  | S02194 | 44 |
|  |  |  | S01573 | 25 |  |  |  |  | S02209 | 74 |
|  |  |  | S01582 | 16 |  |  |  |  | S02234 | 63 |
|  |  |  | S01583 | 12 |  |  |  |  | S02241 | 62 |
|  |  |  | S01584 | 16 |  |  |  |  | S02242 | 45 |
|  |  |  | S01585 | 10 |  |  |  |  | S02243 | 44 |
|  |  |  | S01590 | 23 |  |  |  |  | S02244 | 77 |
|  |  |  | S01593 | 5 |  |  |  |  | S02246 | 11 |
|  |  |  | S01605 | 5 |  |  |  |  | S02248 | 66 |
|  |  |  | S01666 | 21 |  |  |  |  | S02249 | 50 |
|  |  |  | S01731 | 19 |  |  |  |  | S02255 | 35 |
|  |  |  | S01733 | 35 |  |  |  |  | S02256 | 75 |
|  |  |  | S01742 | 27 |  |  |  |  | S02284 | 65 |
|  |  |  | S01746 | 28 |  |  |  |  | S02285 | 43 |
|  |  |  | S02126 | 16 |  |  |  |  | S02286 | 62 |
|  |  |  | S02154 | 33 |  |  |  |  | S02287 | 54 |
|  |  |  | S02162 | 22 |  |  |  |  | S02288 | 61 |
|  |  |  | S02323 | 13 |  |  |  |  | S02289 | 38 |
|  |  |  | S02325 | 15 |  |  |  |  | S02290 | 39 |
|  |  |  | S02326 | 15 |  |  |  |  | S02291 | 46 |
|  |  |  | S02327 | 27 |  |  |  |  | S02293 | 45 |
|  |  |  | S02330 | 24 |  |  |  |  | S02294 | 45 |
|  |  |  | S02331 | 32 |  |  |  |  | S02295 | 71 |
|  |  |  | S02333 | 21 |  |  |  |  | S02296 | 57 |
|  |  |  | S02334 | 16 |  |  |  |  | S02297 | 16 |
|  |  |  | S02335 | 28 |  |  |  |  | S02298 | 51 |
|  |  |  | S02337 | 29 |  |  |  |  | S02299 | 26 |
|  |  |  | S02338 | 18 |  |  |  |  | S02322 | 43 |
|  |  |  | S02339 | 17 |  |  |  |  | S02328 | 68 |
|  |  |  | S02340 | 27 |  |  |  |  | S02342 | 65 |
|  |  |  |  |  |  |  |  |  | S02347 | 37 |
|  |  |  |  |  |  |  |  |  | S02350 | 66 |
|  |  |  |  |  |  |  |  |  | S02351 | 68 |
|  |  |  |  |  |  |  |  |  | S02352 | 44 |
|  |  |  |  |  |  |  |  |  | S02353 | 19 |
|  |  |  |  |  |  |  |  |  | S02354 | 35 |
|  |  |  |  |  |  |  |  |  | S02360 | 77 |
|  |  |  |  |  |  |  |  |  | S02375 | 48 |
|  |  |  |  |  |  |  |  |  | S02376 | 49 |
|  |  |  |  |  |  |  |  |  | S02378 | 44 |
|  |  |  |  |  |  |  |  |  | S02382 | 42 |
|  |  |  |  |  |  |  |  |  | S02397 | 44 |

**Table S2. Chi-Squre tests of correlation study.**

LSD1 and Grade-Crosstabulation

LSD1 and Grade-Chi-Square Tests

LSD1 and Metastasis-Crosstabulation

LSD1 and Metastasis-Chi-Square Tests

LSD1 and KRAS mutation-Crosstabulation

LSD1 and KRAS mutation- Chi-Square Tests

LSD1 and Integrin β3expression-Crosstabulation

LSD1 and Integrin β3expression- Chi-Square Tests

**Table S3. Top 100 down- and up-regulated genes upon LSD1 knockdown in A549.**

|  | TOP 100 down-regulated genes | | |  |  | TOP 100 up-regulated genes | | |
| --- | --- | --- | --- | --- | --- | --- | --- | --- |
|  | gene | logFC | adj.p.value |  |  | gene | logFC | adj.p.value |
| 1 | EDAR | -9.90 | 3.39E-14 |  | 1 | RP11-299H22.5 | 7.99 | 5.79E-06 |
| 2 | MYL9 | -9.03 | 4.00E-17 |  | 2 | RCAN2 | 7.91 | 4.70E-07 |
| 3 | MUC5AC | -8.97 | 2.29E-07 |  | 3 | hsa-mir-7162 | 7.78 | 1.03E-17 |
| 4 | RP5-977B1.11 | -8.47 | 8.19E-14 |  | 4 | C15orf27 | 7.56 | 1.30E-09 |
| 5 | CDH19 | -8.35 | 6.93E-10 |  | 5 | SPTA1 | 7.44 | 2.80E-08 |
| 6 | ZG16B | -8.22 | 9.11E-08 |  | 6 | IL13RA2 | 7.32 | 4.01E-09 |
| 7 | CTA-392C11.1 | -8.20 | 7.07E-07 |  | 7 | HYDIN | 7.25 | 9.28E-07 |
| 8 | RP11-362F19.1 | -8.17 | 2.64E-07 |  | 8 | LRCH2 | 7.23 | 3.50E-38 |
| 9 | PCDH17 | -8.09 | 1.03E-08 |  | 9 | MAGEB18 | 7.19 | 1.42E-06 |
| 10 | KRT19P2 | -7.92 | 2.03E-32 |  | 10 | CCND2 | 7.13 | 4.83E-08 |
| 11 | MUC5B | -7.87 | 6.16E-45 |  | 11 | AC078882.1 | 7.05 | 2.76E-05 |
| 12 | TMEM215 | -7.82 | 5.33E-07 |  | 12 | OR51B4 | 7.01 | 9.98E-06 |
| 13 | PRKCDBP | -7.70 | 3.87E-18 |  | 13 | AC018742.1 | 6.98 | 5.20E-08 |
| 14 | CIB4 | -7.69 | 2.21E-03 |  | 14 | CHL1 | 6.96 | 5.54E-07 |
| 15 | ALOX12P2 | -7.68 | 5.24E-07 |  | 15 | MRC1L1 | 6.96 | 1.51E-07 |
| 16 | U8 | -7.52 | 9.24E-04 |  | 16 | BOLL | 6.94 | 1.12E-06 |
| 17 | MIR492 | -7.51 | 4.26E-28 |  | 17 | MRC1 | 6.90 | 1.09E-06 |
| 18 | RP11-58B17.2 | -7.38 | 8.59E-07 |  | 18 | RBM11 | 6.87 | 1.83E-11 |
| 19 | SPINK6 | -7.34 | 1.91E-07 |  | 19 | JAKMIP2 | 6.75 | 3.26E-10 |
| 20 | AC009299.3 | -7.26 | 6.80E-08 |  | 20 | NAALAD2 | 6.68 | 2.99E-04 |
| 21 | RP11-532E4.2 | -7.22 | 5.32E-11 |  | 21 | FAM13C | 6.61 | 3.53E-06 |
| 22 | GALNTL6 | -7.16 | 6.69E-03 |  | 22 | ZNF610 | 6.61 | 3.17E-06 |
| 23 | ALB | -7.14 | 4.96E-04 |  | 23 | RP11-593F23.1 | 6.57 | 6.85E-05 |
| 24 | DERL3 | -7.13 | 1.48E-07 |  | 24 | RP11-473I1.6 | 6.55 | 2.05E-05 |
| 25 | HOXD3 | -7.08 | 3.75E-06 |  | 25 | MME | 6.51 | 7.79E-05 |
| 26 | MIAT | -7.06 | 1.71E-03 |  | 26 | CDK14 | 6.49 | 5.20E-05 |
| 27 | ARMCX1 | -7.02 | 9.81E-16 |  | 27 | OXGR1 | 6.46 | 4.33E-06 |
| 28 | VIL1 | -6.99 | 1.12E-04 |  | 28 | CNTN5 | 6.44 | 1.16E-10 |
| 29 | APBB1IP | -6.98 | 5.12E-16 |  | 29 | CTD-2353F22.1 | 6.42 | 2.76E-05 |
| 30 | CES1P1 | -6.96 | 5.48E-04 |  | 30 | GLB1L3 | 6.41 | 2.65E-05 |
| 31 | CA9 | -6.95 | 2.74E-08 |  | 31 | PHACTR1 | 6.40 | 6.05E-06 |
| 32 | CES1 | -6.93 | 3.42E-12 |  | 32 | CACNA1B | 6.37 | 1.50E-08 |
| 33 | HOXD4 | -6.87 | 5.14E-06 |  | 33 | NLGN1 | 6.32 | 5.10E-04 |
| 34 | SLC44A4 | -6.86 | 1.05E-02 |  | 34 | AC007405.6 | 6.30 | 2.46E-03 |
| 35 | HHIP | -6.73 | 1.70E-04 |  | 35 | HYDIN2 | 6.28 | 2.10E-04 |
| 36 | SLC1A7 | -6.68 | 2.12E-03 |  | 36 | CAPN6 | 6.27 | 3.43E-07 |
| 37 | KRT19 | -6.68 | 1.84E-33 |  | 37 | DMGDH | 6.27 | 8.19E-03 |
| 38 | RP1-276N6.2 | -6.67 | 2.08E-05 |  | 38 | HRG | 6.27 | 9.77E-05 |
| 39 | HTRA3 | -6.63 | 2.10E-06 |  | 39 | EDNRB | 6.26 | 1.62E-02 |
| 40 | EDN3 | -6.63 | 1.77E-03 |  | 40 | SCML2P1 | 6.26 | 2.51E-04 |
| 41 | LAYN | -6.61 | 1.22E-08 |  | 41 | GPC3 | 6.25 | 2.30E-05 |
| 42 | HOXD-AS2 | -6.61 | 9.98E-05 |  | 42 | TRAT1 | 6.22 | 5.53E-04 |
| 43 | TFF1 | -6.57 | 4.41E-13 |  | 43 | PYHIN1 | 6.21 | 1.44E-05 |
| 44 | HGF | -6.56 | 1.86E-02 |  | 44 | FLI1 | 6.18 | 7.83E-04 |
| 45 | AL354933.1 | -6.53 | 2.27E-42 |  | 45 | OR51B5 | 6.16 | 6.06E-05 |
| 46 | KRT19P1 | -6.51 | 1.69E-43 |  | 46 | TRBV20-1 | 6.14 | 1.55E-04 |
| 47 | ADAMTSL3 | -6.47 | 8.63E-05 |  | 47 | ERC2 | 6.14 | 9.19E-04 |
| 48 | MAOB | -6.40 | 3.80E-05 |  | 48 | SERPINI2 | 6.12 | 2.10E-04 |
| 49 | PTPRN | -6.40 | 3.37E-04 |  | 49 | AC104389.28 | 6.12 | 1.96E-10 |
| 50 | TMPRSS3 | -6.38 | 2.21E-03 |  | 50 | FOLR3 | 6.11 | 3.80E-03 |
| 51 | TFF3 | -6.36 | 2.39E-03 |  | 51 | TMPRSS11D | 6.10 | 1.89E-04 |
| 52 | CXCL6 | -6.36 | 2.82E-03 |  | 52 | WIF1 | 6.09 | 8.78E-10 |
| 53 | KCNJ15 | -6.34 | 5.12E-03 |  | 53 | FGF13 | 6.08 | 5.02E-14 |
| 54 | GABRA3 | -6.34 | 3.47E-09 |  | 54 | PCDH11X | 6.07 | 2.30E-02 |
| 55 | MYO7B | -6.32 | 1.41E-04 |  | 55 | LECT2 | 6.06 | 1.35E-03 |
| 56 | BPIFB1 | -6.30 | 8.59E-07 |  | 56 | ESM1 | 6.05 | 3.11E-04 |
| 57 | SERPINA1 | -6.29 | 2.34E-03 |  | 57 | C5orf46 | 6.05 | 1.04E-03 |
| 58 | ENTPD2 | -6.29 | 2.06E-06 |  | 58 | RP3-326I13.1 | 6.05 | 4.24E-34 |
| 59 | RP11-371I1.2 | -6.28 | 1.01E-09 |  | 59 | SSTR1 | 6.04 | 3.40E-04 |
| 60 | ANKS4B | -6.24 | 2.58E-10 |  | 60 | SOST | 6.03 | 2.22E-03 |
| 61 | MUC2 | -6.22 | 9.69E-05 |  | 61 | LIX1 | 6.02 | 2.46E-03 |
| 62 | WDR86 | -6.19 | 1.78E-03 |  | 62 | CTC-504A5.1 | 6.01 | 2.06E-04 |
| 63 | CHRDL2 | -6.17 | 1.86E-04 |  | 63 | CTD-3162L10.1 | 6.00 | 1.50E-04 |
| 64 | ST6GAL2 | -6.15 | 1.44E-33 |  | 64 | CAMK4 | 6.00 | 7.42E-09 |
| 65 | SLC2A10 | -6.13 | 3.96E-04 |  | 65 | ATP8A2 | 5.99 | 3.38E-04 |
| 66 | RPS6KL1 | -6.09 | 2.20E-04 |  | 66 | LINC00668 | 5.99 | 8.65E-05 |
| 67 | RP11-116O11.1 | -6.09 | 5.69E-04 |  | 67 | GPR85 | 5.99 | 8.52E-04 |
| 68 | SMIM10 | -6.08 | 1.57E-02 |  | 68 | CDH12 | 5.98 | 3.98E-09 |
| 69 | LDLRAD4 | -6.07 | 2.68E-04 |  | 69 | PEX5L | 5.97 | 3.75E-04 |
| 70 | CSMD3 | -6.05 | 1.03E-03 |  | 70 | C8orf34 | 5.96 | 4.54E-04 |
| 71 | ADAMTSL1 | -5.92 | 5.65E-03 |  | 71 | LRRC7 | 5.94 | 1.64E-04 |
| 72 | LHFPL4 | -5.89 | 3.23E-02 |  | 72 | RP11-89M20.2 | 5.94 | 2.41E-03 |
| 73 | AP000439.3 | -5.88 | 6.64E-11 |  | 73 | NELL2 | 5.94 | 1.70E-04 |
| 74 | TRPC6P | -5.83 | 6.37E-04 |  | 74 | TRAV16 | 5.92 | 1.37E-04 |
| 75 | ART1 | -5.82 | 3.33E-02 |  | 75 | RP11-299H22.6 | 5.91 | 1.06E-02 |
| 76 | RP11-530N7.3 | -5.82 | 4.57E-03 |  | 76 | FREM1 | 5.91 | 1.06E-02 |
| 77 | EPS8L3 | -5.81 | 1.24E-05 |  | 77 | AC010296.1 | 5.90 | 5.97E-04 |
| 78 | PODN | -5.81 | 6.33E-03 |  | 78 | AMPH | 5.87 | 3.14E-03 |
| 79 | MIXL1 | -5.78 | 3.96E-03 |  | 79 | RP11-267C16.1 | 5.87 | 2.34E-04 |
| 80 | AKR1C2 | -5.77 | 1.18E-10 |  | 80 | GRM2 | 5.85 | 2.89E-03 |
| 81 | SERPING1 | -5.76 | 5.09E-03 |  | 81 | MADCAM1 | 5.85 | 2.89E-03 |
| 82 | SVOPL | -5.72 | 2.16E-02 |  | 82 | KIF1A | 5.84 | 1.16E-02 |
| 83 | AC114812.5 | -5.72 | 8.78E-03 |  | 83 | TENM4 | 5.83 | 3.50E-04 |
| 84 | CASC16 | -5.72 | 2.18E-02 |  | 84 | NETO1 | 5.80 | 6.44E-04 |
| 85 | PTGDS | -5.68 | 4.31E-02 |  | 85 | SATB1 | 5.79 | 4.25E-04 |
| 86 | BPIFA2 | -5.68 | 1.64E-02 |  | 86 | VIT | 5.79 | 1.39E-03 |
| 87 | RP11-790I12.1 | -5.66 | 2.65E-03 |  | 87 | TFEC | 5.79 | 4.26E-04 |
| 88 | FAIM2 | -5.64 | 1.37E-02 |  | 88 | CCDC102B | 5.79 | 4.26E-04 |
| 89 | RP11-506N2.1 | -5.62 | 1.48E-03 |  | 89 | EPYC | 5.78 | 6.28E-03 |
| 90 | RP11-166D19.1 | -5.62 | 3.68E-02 |  | 90 | LRMP | 5.78 | 6.28E-03 |
| 91 | SAMSN1 | -5.61 | 4.47E-02 |  | 91 | LAMA1 | 5.78 | 3.22E-04 |
| 92 | WNT6 | -5.59 | 3.02E-02 |  | 92 | SLCO1A2 | 5.78 | 5.04E-03 |
| 93 | AC124789.1 | -5.56 | 5.47E-03 |  | 93 | STMN4 | 5.75 | 2.38E-03 |
| 94 | AACSP1 | -5.55 | 3.93E-03 |  | 94 | KIT | 5.75 | 3.71E-04 |
| 95 | GUCA2B | -5.54 | 1.24E-02 |  | 95 | AK9 | 5.74 | 2.64E-02 |
| 96 | LYL1 | -5.51 | 4.81E-03 |  | 96 | DTNA | 5.74 | 1.29E-02 |
| 97 | FBLN2 | -5.48 | 3.99E-03 |  | 97 | ANKRD62 | 5.73 | 4.48E-03 |
| 98 | RP11-632K21.3 | -5.45 | 5.59E-03 |  | 98 | LEF1 | 5.73 | 7.50E-04 |
| 99 | TRPC6 | -5.44 | 2.31E-05 |  | 99 | TMEM52B | 5.72 | 3.38E-10 |
| 100 | SNAP25-AS1 | -5.38 | 1.73E-02 |  | 100 | CXorf57 | 5.72 | 1.14E-15 |

**Table S4.** Primer sets used in this study.

| **Primers** |  |
| --- | --- |
| AGR2-F | ggtgggtgaggaaatccag |
| AGR2-R | gtaggagagggccacaagg |
| AKR1B10-F | aaagcaacgttcttggatgc |
| AKR1B10-R | tggaagtggctgaaattgg |
| AKR1C1-F | catgcctgtcctgggattt |
| AKR1C1-R | agaatcaatatggcggaagc |
| AKR1C3-F | cattggggtgtcaaacttca |
| AKR1C3-R | ccggttgaaatacggatgac |
| APOC1-F | gccttggataagctgaagga |
| APOC1-R | gaaatgtctctgaaaaccactcc |
| AQP3-F | cgcctttttacagcccttg |
| AQP3-R | caggagtggggacacgat |
| BEX2-F | gatgcagaaaatggtggtttg |
| BEX2-R | cctctttggactccattactcc |
| BMP6-F | acatggtcatgagctttgtga |
| BMP6-R | actctttgtggtgtcgctga |
| CES1-F | tggtgtcccatctgtgattg |
| CES1-R | gaagcttggacggtactgaaa |
| CXCL1-F | tcctgcatcccccatagtta |
| CXCL1-R | cttcaggaacagccaccagt |
| CYP3A7-F | caaacttggccgtggaaa |
| CYP3A7-R | agtccatgtgtacgggttcc |
| DOCK3-F | gatggacacgtatatccagaagc |
| DOCK3-R | acttcaaacagcggatgagc |
| FGFR2-F | ctcactctcacaaccaatgagg |
| FGFR2-R | aactaggtgaatactgttcgagagg |
| FOXA1-F | agggctggatggttgtattg |
| FOXA1-R | accgggacggaggagtag |
| HCN4-F | ggtgtccatcaacaacatgg |
| HCN4-R | gccttgaagagcgcgtag |
| HHIP-F | tcctcaatgtgaacaagtggac |
| HHIP-R | ggacagggtaaaggtgtttcat |
| HLA-B-F | cctacctggagggcgagt |
| HLA-B-R | ggtgggtcacgtgtgtcttt |
| HS6ST2-F | agacccggaacacatctaagag |
| HS6ST2-R | tcaagtaccgggacactgg |
| HSPA6-F | ggataaaaagcccgtggaa |
| HSPA6-R | gctctccctgcggtttct |
| IGFBP3-F | aacgctagtgccgtcagc |
| IGFBP3-R | cggtcttcctccgactcac |
| IRS2-F | ttcttgtcccaccacttgaa |
| IRS2-R | ctgacatgtgacatcctggtg |
| ITGB2-F | cagcaatgtggtccaactca |
| ITGB2-R | gagggcgttgtgatccag |
| ITGB3-F | cgctaaatttgaggaagaacg |
| ITGB3-R | gaaggtagacgtggcctcttt |
| ITGB4-F | tcagcctctctgggacctt |
| ITGB4-R | tccttatccacacggacaca |
| KCNE4-F | caaaacttgggacaaactgtca |
| KCNE4-R | gtgctgttcagaggctccat |
| LAMP3-F | atttgaccgtctcagatccag |
| LAMP3-R | cttgaaggaatgcccgact |
| LBP-F | gtggacatgtcgggagactt |
| LBP-R | ggactcaatctggttgtggaa |
| LGALS3-F | cttctggacagccaagtgc |
| LGALS3-R | aaaggcaggttataaggcacaa |
| LSD1-F | cccttaagcactgggatcag |
| LSD1-R | acacgagtagccattccttactg |
| MUC5B-F | cgaggtcaacatcaccttctg |
| MUC5B-R | gggcctctgctgagtacttg |
| NKX2.1-F | tcatttgttggcgactgg |
| NKX2.1-R | tgctttggactcatcgacat |
| PP1R14C-F | ttacaggaagctcttgtagactgc |
| PP1R14C-R | gcctcttatccgagaaagca |
| RAB38-F | gcctgcagctctgggata |
| RAB38-R | ttccactttgccactgctt |
| RAB39B-F | gagccaggaaaacgcatc |
| RAB39B-R | taggcgcgagtgatggat |
| RAB3B-F | tgacatcaccaatgaagagtcc |
| RAB3B-R | tgtcccaggagtaggtcttga |
| RAB9B-F | acccttctacaggggagca |
| RAB9B-R | gctctgccgatcatccac |
| RALBP1-F | tctaacatggccacgatgc |
| RALBP1-R | gatgtaaacaattcaaaagaaactcct |
| RASGRP1-F | gagccaaagatctgctccat |
| RASGRP1-R | ggtccgatccttactctcctc |
| RASGRP3-F | ccactgaattggcagaaaaac |
| RASGRP3-R | tcgaaattcattgcagctttc |
| RBP4-F | ccagaagcgcagaagattg |
| RBP4-R | tttctttctgatctgccatcg |
| SOX9-F | gtacccgcacttgcacaac |
| SOX9-R | tctcgctctcgttcagaagtc |
| SPP1-F | gagggcttggttgtcagc |
| SPP1-R | caattctcatggtagtgagttttcc |
| STC1-F | aggcggagcagaatgactc |
| STC1-R | gttgaggcaacgaaccactt |
| TFF1-F | cccctggtgcttctatccta |
| TFF1-R | gatccctgcagaagtgtctaaaa |
